# The Effect of Fluoroquinolones on Aortic Aneurysm and Dissection

**DOI:** 10.7759/cureus.74740

**Published:** 2024-11-29

**Authors:** Luis A Morales-Ojeda

**Affiliations:** 1 Vascular Surgery, University of Colorado Anschutz Medical Center, Colorado, USA

**Keywords:** aortic aneurysm, aortic dissection, broad spectrum antibiotics, fluoroquinolones, general and vascular surgery, matrix metalloproteinases

## Abstract

Fluoroquinolones (FQs) are a widely prescribed class of antibiotics including ciprofloxacin, levofloxacin, and ofloxacin. They are commonly used to treat a variety of infections worldwide. Known for their broad-spectrum antimicrobial activity, as well as excellent pharmacokinetics and bioavailability, the use of FQs has risen significantly. While generally well-tolerated, research has indicated that FQs may aggravate collagen-related disorders. These antibiotics increase the activity of matrix metalloproteinases (MMPs), particularly MMP-2 and MMP-9, which degrade type I collagen, weakening the structural integrity of tendons. Some studies have demonstrated the association between FQs and the risk of aortic aneurysm or dissection. Given the life-threatening nature of these conditions, these findings are particularly concerning.

## Editorial

Antibiotics are essential in treating infections, including those associated with aortic aneurysms. Mycotic aneurysms require effective antimicrobial therapy to prevent severe complications and support surgical interventions. While fluoroquinolones (FQs) are valued for their broad-spectrum activity and efficacy, emerging evidence has linked their use to an increased risk of aortic aneurysm and dissection, raising concerns about their safety in specific populations. FQs have been associated with adverse effects on connective tissue, including tendon disorders, which are thought to result from the disruption of collagen metabolism [1]. Given the structural similarities between tendons and the aortic wall, this class of antibiotics has been implicated in vascular complications, particularly in individuals with underlying risk factors such as advanced age or pre-existing connective tissue disorders.

Substantial evidence supports the association between FQ use and aortic complications. For instance, Garg et al. conducted a large cohort study, demonstrating a significantly increased risk of aortic aneurysm or dissection among FQ users compared to non-users [2]. Garg et al. used a propensity score-matched design, which provided more support to their conclusions. Similarly, Newton et al. identified a short-term increase in the risk of aortic aneurysm following FQ exposure, suggesting a temporal link between use and adverse vascular outcomes [3]. Dong et al. extended these findings by demonstrating a dose-response relationship, where higher cumulative exposure to FQs was associated with greater risk [4]. However, it should be noted that the risk of aortic dissection or aneurysm is influenced by other clearly identified risk factors, many of which are not modifiable. Further research must focus on establishing which patient profiles such as advanced age, pre-existing connective tissues disorders, or specific comorbidities are most closely related to an elevated risk of dissection or aneurysm in the context of FQ use.

Despite this evidence, some researchers have failed to confirm these associations. For instance, Pasternak et al. explored the risk of aortic complications in a large cohort and found a weaker association between FQ use and aneurysms, suggesting the presence of confounding factors, such as the severity of infection, could explain the results [5]. These conflicting findings highlight the need for further research to clarify the exact risk. Notably, Prashanth Rawla et al. confirmed a positive association between FQs and the development of aortic aneurysm or dissection. Their data suggest that this association is primarily driven by the risk of aortic aneurysm. When considering FQ therapy clinicians should carefully evaluate individual patient profiles [5]. The implications for clinical practice are significant. Aortic aneurysm and dissection are potentially fatal conditions, and the risks associated with FQs must be carefully weighed against their potential benefits [5]. High-risk groups, including older population, and patients with connective tissue disorders, may benefit from alternative antibiotics when possible.

Furthermore, clinicians should remain vigilant in monitoring patients for early signs of vascular complications and educating them about potential risks. The association between FQs and aortic complications underscores the broader challenge of balancing the benefits of antibiotics with patient safety [5]. As research continues advancing, our understanding and informed decision making must be tailored to individuals' risk profiles. In the meantime, the adoption of conservative prescribing practices can help mitigate unnecessary exposure to high-risk medications. 
